# Addition of metformin to non‐small‐cell lung cancer patients with or without diabetes

**DOI:** 10.1111/1759-7714.14756

**Published:** 2022-11-30

**Authors:** Hua‐Fen Chen, Rong Lin, Chen‐Xiong Hsu

**Affiliations:** ^1^ Division of Endocrinology and Metabolism, Department of Internal Medicine Far Eastern Memorial Hospital New Taipei City Taiwan; ^2^ School of Medicine and Department of Public Health College of Medicine, Fu Jen Catholic University New Taipei City Taiwan; ^3^ Division of General Internal Medicine, Department of Internal Medicine Far Eastern Memorial Hospital New Taipei City Taiwan; ^4^ Division of Radiation Oncology, Department of Radiology Far Eastern Memorial Hospital New Taipei City Taiwan

To the Editor,

We read with interest the recently published article by Yang et al.^1^ investigating patients with metastatic non‐small‐cell lung cancer (NSCLC) treated with immune checkpoint inhibitors. The authors concluded that patients with diabetes concurrently taking metformin or a combination of metformin and dipeptidyl peptidase‐4 inhibitors had a significantly higher objective response rate (ORR) and progression‐free survival (PFS) compared to those without diabetes. We still have some concerns about this article.

Metformin, an antidiabetic agent, is proposed to suppress the tumor growth through inhibition of complex I of the mitochondria oxidative phosphorylation chain, activation of AMP‐activated protein kinase, suppression of the insulin‐like growth factor‐I receptor and PI3K/AKT/mTOR pathways, and stimulation of the immune system.^2^ It is the first‐line agent for most patients with diabetes, but Yang et al.^1^ did not evaluate the diabetes complications, comorbidities or report the status of diabetic control in their study. Yang and colleagues also excluded those patients taking other oral hypoglycemic agents or insulin use, which might have eliminated more complicated patients with diabetes. Those patients who take metformin might have been free of more severe clinical conditions such as hepatic impairment, heart failure or chronic kidney disease which are common contraindications to metformin use,^3^ and it is not clear whether patients with diabetes in lesser comorbidities might have been associated with higher ORR or PFS.

Two recently published studies demonstrated that patients with diabetes had lower overall survival compared to those without diabetes in stage IIIB‐IV NSCLC or squamous cell lung cancer after propensity score matching analysis.^4^, ^5^ Diabetes complications significantly impacted the overall survival of patients with lung cancer, and adapted diabetes complications severity index (aDCSI) score ≥2 is an independent prognostic factor,^5^ whereas the severity of patients with diabetes was not reported in the study by Yang et al.^1^

In recent randomized clinical trials, the addition of metformin to chemoradiotherapy in locally advanced NSCLC patients without diabetes showed no additional survival benefit, worse treatment efficacy, and increased toxicities.^6^, ^7^ We appreciate Yang and colleagues for their contribution and insights to the study. Future trial designs that consider the impact of metformin use, status of diabetic control, complications, comorbidities, and severity of diabetes on ORR and PFS in patients with metastatic NSCLC are necessary to clarify these issues.

## AUTHOR CONTRIBUTIONS

All authors contributed to the article and approved the submitted version.

## FUNDING INFORMATION

This letter received no financial support.

## CONFLICT OF INTEREST

The authors declare no conflict of interest.
